# The Effect of Substances of Plant Origin on the Thermal and Thermo-Oxidative Ageing of Aliphatic Polyesters (PLA, PHA)

**DOI:** 10.3390/polym10111252

**Published:** 2018-11-12

**Authors:** Anna Masek, Malgorzata Latos-Brozio

**Affiliations:** Institute of Polymer and Dye Technology, Lodz University of Technology, ul. Stefanowskiego 12/16, 90-924 Lodz, Poland; malgorzata.latos@edu.p.lodz.pl

**Keywords:** polyester, flavonoids, stabilizers, ageing, crystallization, thermo-oxidative

## Abstract

The stabilization efficiency of flavonoids (rutin and hesperidin) in polyester (polylactide (PLA) and polyhydroxyalkaonate (PHA)) composites under oxygen at high temperature was investigated. The polymer was homogenized with three antioxidants then processed by extrusion. The effects of stabilizers on the following physicochemical properties were investigated: melt flow, Vicat softening temperature, surface energy, and color change (Cie-Lab space). The aim of this study was to improve the stability of aliphatic polyesters by extending and controlling their lifetime. Differential Scanning Calorimetry DSC and Thermogravimetric analysis DTG methods were used to confirm the stabilizing effects (the inhibition of oxidation) of flavonoids (rutin and hesperidin) on the ageing process of biodegradable polymers. The levels of migration of plant antioxidants from PLA and PHA were determined and compared to the industrial stabilizer (Chimassorb 944 UV absorber). Based on this study, a comparable-to-higher efficiency of the proposed flavonoids for the stabilization of polyesters was found when compared to the commercial stabilizers. Thus, in the future, natural plant-derived substances may replace toxic hindered amines, which are commonly used as light stabilizers (HALS—Hindered Amine Light Stabilizers) in the polymer industry.

## 1. Introduction

Biopolymers, polymers from renewable raw materials, have recently become the most attractive alternative to synthetic non-degradable petro-polymers. The extension of the shelf life of food packed in polymers can also be achieved by the addition of antioxidants to the polymer resin. Environmental protection concerns and the rising cost of oil have pushed biodegradable polymers into world markets [1,2,3,4,5,6]. Unfortunately, aliphatic biopolymers such as polylactide (PLA) are characterized by poor resistance to climatic factors mainly due to the presence of polar ester groups that increase their susceptibility to hydrolysis. Apart from biodegradation, PLA can undergo photodegradation and hydrolytic degradation. However, new polymers that are being explored should have stability for some time. Despite this disadvantage, PLA is considered to be a green polymer and a polymer of the 21st century. Polylactide is characterized by its ease of processing, good mechanical properties (particularly Young’s modulus), a glass transition temperature higher than room temperature, and higher transparency, all of which indicate that PLA is a promising material for packaging and other industrial applications. PLA has good physical and mechanical properties, making it possible to replace petrochemical thermoplastics. Additionally, PLA processing can be carried out using existing machines [7,8,9,10,11,12,13,14,15,16,17,18,19,20,21]. Biodegradable polymers obtained from microbial fermentation, especially polyhydroxyalkaonates (PHAs), are biodegradable, biocompatible, and thermo-processable with flexible strengths. Aliphatic polyesters are products which can be degraded by enzymatic activities of various microorganisms, and the degradation products are non-toxic. PHAs have found applications in the form of packaging materials including films, boxes, and coatings. The biodegradable PHA products can reduce the disposing of packaging waste that is burdening life worldwide through a substantial reduction in non-degradable polyolefins waste [22,23,24,25,26].

The addition of flavonoids results in the stabilization of the polymer during processing. This solution also delays the oxidation of the lipid components of nutritional products. The antioxidant is added to the polymer via the following mechanisms: Diffusion of antioxidant molecules from the interior of the polymer phase to its surface; oxidation or evaporation of the antioxidant from the polymer surface; and migration or antioxidant sorption onto the surface of the packaged product. In the field of polymer technology, natural antioxidants are increasingly becoming a green alternative to compounds such as hindered amine light stabilizers (HAS), phosphate derivatives, and synthetic polyphenols. There have been studies reported in the literature concerning the use of natural flavonoids for the stabilization of polymers, but the research primarily concerns polyolefins, which are commonly used in packaging. Flavonoids act as antioxidants and interact directly with free radicals or indirectly cause an increase in free radical scavenging, leading to compounds with a lowered reactivity. Flavonoids have the ability to chelate pro-oxidation metals, which prevents the formation of reactive hydroxyl radicals in cells and inhibits or enhances the activity of many different enzymes. The benefit of mixing free radicals with natural antioxidants is that the antioxidants will react with free radicals, protecting cells from free radical damage and interrupting free radical reactions [27,28,29,30,31,32,33,34,35,36,37]. Therefore, in our research, we decided to combine two ecological materials derived from renewable raw materials, namely, aliphatic polyesters and natural flavonoid antioxidants. This solution seems to be very beneficial, both in terms of environmental protection and human health [38,39,40,41,42].

## 2. Materials and Methods

### 2.1. Reagents

**Figure 1:**
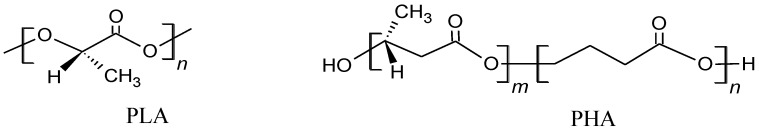
Chemical composition of polylactide (PLA) and polyhydroxyalkanoate (PHA).

The subjects of the study were polylactide (PLA) Ingeo^TM^ Biopolymer 4043D from Nature Works^TM^ (Minnetonka, MN, USA) with the following properties: *T*_g_ = 55–60 °C, *T*_m_ = 145–160 °C, and Melt Flow Index MFI = 6 g/10 min and polymer P(3,4HB) 2001 from the polyhydroxyalkanoate group of polymers (PHA) with the following properties: P(3,4HB) containing 12 mol% 4-hydroxybutyrate, the average *M*_w_ was approximately 520 kDa, MVR = 15–20 g/10 min, (assay conditions: temperature 170 °C, nominal load 2.16 kg), and a density of 1.25 g/cm^3^. PHA was obtained from Simag Holdings LTD (Hong Kong, China) (Figure 1).

**Figure 2:**
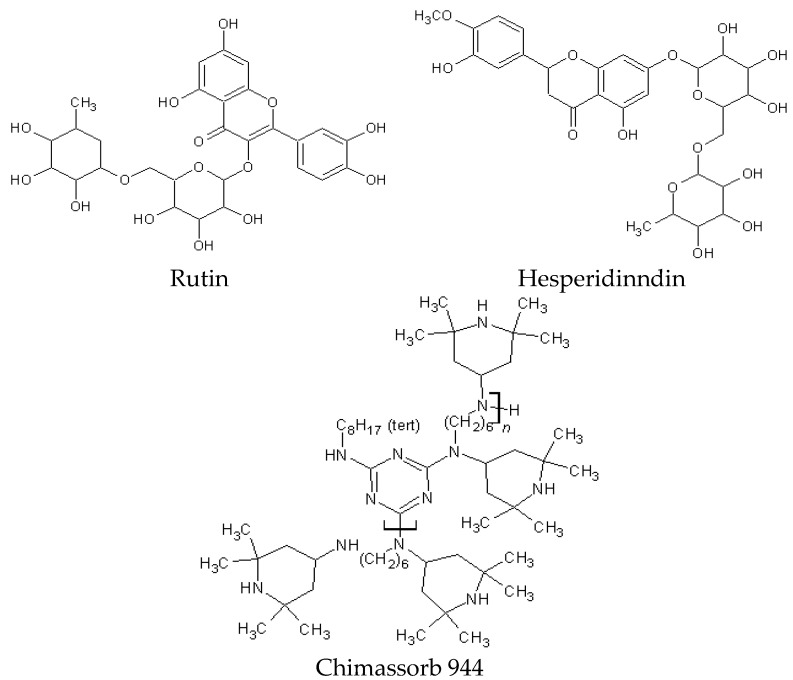
Chemical composition of rutin, hesperidin, and Chimassorb 944.

Different flavonoids were used as stabilizer additives at a concentration of 1 phr (parts per hundred rubber) for PLA (100 phr) and PHA (100 phr). Pure rutin powder (≥94%, HPLC) and hesperidin (Hesperetin 7-rhamnoglucoside, ≥80%) were obtained from a commercial source (Sigma-Aldrich, Steinheim, Germany) and used as received. The oligomeric hindered amine light stabilizer (HAS), Chimassorb 944 (Poly[[6-[(1,1,3,3-tetramethylbutyl)amino]-1,3,5-triazine-2,4-diyl][(2,2,6,6-tetramethyl-4-piperidinyl)imino]-1,6 hexanediyl[(2,2,6,6-tetramethyl-4-piperidinyl)imino]]), was obtained from Ciba (Basel, Switzerland) (Figure 2).

### 2.2. Methods for the Preparation of PLA and PHA Composites with Antioxidants

The granules of each of the PLA and PHA polyesters with a specific antioxidant were subjected to extrusion in a Brabender extruder working in the horizontal position with a screw diameter of Ø 25. All samples were produced under specific extrusion conditions: Polylactide polymer (PLA) samples were prepared at 180 °C, while polyhydroxyalkanoate (PHA) samples were extruded at 160 °C.

### 2.3. Measurement Methods

#### 2.3.1. Thermal Analysis of PLA and PHA Composites with Antioxidants

The crystallized specimens were characterized by using temperature-modulated DSC (TA 2920; TA Instruments, Greifensee, Switzerand) at a heating rate of 5 °C/min with a 60 s heating/cooling cycle for the modulation period and an amplitude of ±0.769 °C to determine the glass transition (*T*_g_) crystallization temperature (*T*_c_) and the melting temperature (*T*_m_). The DSC was calibrated with indium before use. For the measurement of *T*_c_, we used amorphous samples after quenching from the melt state (~200 °C).

Thermal decomposition (TG) was studied by using a Mettler Toledo Thermobalance (TA Instruments, Greifensee, Switzerand). Samples of approximately 5 mg were placed in aluminum pans and heated from 20 to 650 °C under a dynamic flow of nitrogen (50 mL/min). Five heating rates (5 °C/min) were used.

The Vicat softening temperature was determined with a D-Vicat.HDT/3/300/FA material testing device (Peter Huber Kältemaschinenbau GmbH, Offenburg, Germany). The temperature was standardized in ISO 306 and ASTM D 1525. This was the temperature at which a needle with a 1 mm^2^ cross-sectional area at a predetermined load penetrated the test sample to a depth of 1 mm at a defined rate of temperature rise. The measurement parameters were as follows: load = 10 N, inductive displacement gauge resolution = 0.001 mm, temperature gradient = 120 °C/h, initial temperature = 45 °C, final temperature = 150 °C, and displacement range was up to 15 mm.

The melt flow index (MFI) of the extruded pellets was determined using a PIN 7026 melt flow instrument (CEAST, Planegg, Germany). The melt index was measured at 190 °C with a mass of 2.16 kg according to ASTM D1238. The pellets were dried under vacuum at 80 °C for 12 h before the MFI tests. The test was performed in accordance with ISO 1133D using the MELT-FLOW-INDEX TESTER MeltFloWon plus (CEAST, Planegg, Germany).

#### 2.3.2. Ageing of PLA and PHA Composites with Antioxidants

Thermo-oxidation ageing characteristics were determined according to the standard PN-82/C-04216. Samples were exposed to air at an elevated temperature (383 K) for 10 days in a dryer with thermo-circulation.

UV ageing was performed using a UV 2000 apparatus from Atlas. The measurement lasted 288 h and consisted of two alternately repeating segments with the following parameters: daily segment (radiation intensity = 0.7 W/m^2^, temperature = 60 °C, and duration = 8 h) and night segment (no UV radiation, temperature = 50 °C, and duration = 4 h).

Weathering was carried out using a Weather-Ometer (Atlas; Ci 4000, Chicago, IL, USA). The test was based on two variable segments simulating day and night conditions, and the samples were subjected to two different cycles: daily cycle (radiation intensity = 0.4 W m^−2^, temperature = 60 °C, duration = 240 min, humidity = 80%, and rain water on) and night cycle (no radiation, temperature = 50 °C, humidity = 60%, and duration = 120 min).

#### 2.3.3. Surface Properties and Color Characteristics of PLA and PHA Composites with Antioxidants

The color of the obtained materials was measured using a CM-3600d spectrophotometer (Konica Minolta Sensing, Inc., Osaka, Japan). The wavelength range was 360–740 nm, and the change in color dE*ab was calculated as follows:

(1) $$\mathrm{dE}*\mathrm{ab}=\sqrt{\left(\Delta a^{2})+(\Delta b^{2})+(\Delta L^{2}\right)}$$

where L is 100, which represents an ideally reflecting diffuser. The minimum value for L is zero, which corresponds to the color black. The a* and b* axes were not limited to special units. A positive value for a* is red; a negative a* is green; a positive b* is yellow; and a negative b* is blue.

The whiteness index is a numerical indicator used to indicate the degree of whiteness. In the CIE Lab color space, two of the axes are perceptually orthogonal to lightness. Hue can be calculated together with chroma, transforming coordinates a and b from rectangular form to polar form. Hue is angular component of the polar representation whereas chroma is the radial component.

(2) $$\mathrm{Whiteness}\mathrm{index}:\mathrm{Wi}=100-\sqrt{{\left(100-L\right)}^{2}+a^{2}+b^{2}}$$

(3) $$\mathrm{Chroma}:C_{\mathrm{ab}}^{*}=\sqrt{a^{2}+b^{2}}$$

Hue:

h*_ab_ = arc tg (b/a); where a > 0 and b > 0 (4)

h*_ab_ = 180 + arc tg (b/a); where a < 0 and b > 0 (5)

In order to measuring the surface free energy of PLA and PHA composites with antioxidants, the contact angle was determined using a metallographic microscope. Plate samples of PLA and PHA were placed in drops of distilled water and, for subsequent measurements, in droplets of water and diiodomethane using a microsyringe. The contact angle was calculated from the mean of 5 measurements. Measurements were made for samples before ageing and after UV ageing.

Based on measurements of contact angles for four measuring fluids (with different polarities), the free surface energy of the solids (γ_s_) was determined. Free surface energy is the sum of two components—the dispersive (γ_S_^D^) and non-dispersive (γ_S_^P^) effects:

γ_s_ = γ_S_^D^ + γ_S_^P^ (6)

where γ_s_ is the free surface energy (mJ/m^2^), γ_S_^D^ is the dispersion component of the free energy of the tested materials (mJ/m^2^), and γ_S_^P^ is the polar component of the free energy of the tested materials (mJ/m^2^).

In the Owens-Wendt model, the following equations were used for the dispersion component and the polar component:

(7) $${\left(\gamma_{S}^{P}\right)}^{0,5}=\frac{\gamma_{w}\cdot \left({\cos\theta }_{w}+1\right)-2\cdot \sqrt{\gamma_{w}^{D}\cdot \gamma_{S}^{D}}}{2\cdot \sqrt{\gamma_{w}^{P}}}$$

(8) $${\left(\gamma_{S}^{D}\right)}^{0,5}=\frac{\gamma_{D}\cdot \left({\cos\theta }_{D}+1\right)-\sqrt{\frac{\gamma_{D}^{P}}{\gamma_{w}^{P}}}\cdot \gamma_{w}\cdot \left({\cos\theta }_{w}+1\right)}{2\cdot \left[\sqrt{\gamma_{D}^{D}}-\sqrt{\gamma_{D}^{P}\cdot \frac{\gamma_{w}^{D}}{\gamma_{w}^{P}}}\right]}$$

where: $\gamma_{w}$ = water surface free energy (=72.6 mJ/m^2^)

$\gamma_{w}^{D}$_,_$\gamma_{w}^{P}$ = water dispersion component (=21.6 mJ/m^2^) and water polar component (=51.0 mJ/m^2^)

$\gamma_{D}$ = diiodomethane surface free energy (=50.8 mJ/m^2^)

$\gamma_{D}^{D}$_,_$\gamma_{D}^{P}$ = diiodomethane dispersion component (=48.5 mJ/m^2^) and polar component (=2.3 mJ/m^2^)

Specific migration tests were performed according to the European Standard EN 13130. The release of stabilizers from the polyesters was studied in one simulant (model solutions): ethanol at a temperature of 25 °C. Sheets with an area of 5 cm^2^ and a thickness of 4 mm were immersed completely in 10 mL of ethanol and incubated at the chosen temperature for 14 days. A volume of 2 mL of the model solution (ethanol) in contact with polyester stabilized with flavonoids was taken at 2, 4, 6, 8, 10, 12, and 14 days of incubation in order to follow the release of the additive with time. The solutions were scanned from 190 to 1100 nm in a UV-spectrophotometer (Evolution 220, Thermo Fisher Scientific, Waltham, MA, USA).

## 3. Results and Discussion

### 3.1. Thermal Analysis (Differential Scanning Calorimetry and Thermogravimetry) of PLA and PHA Composites with Antioxidants

**Figure 3:**
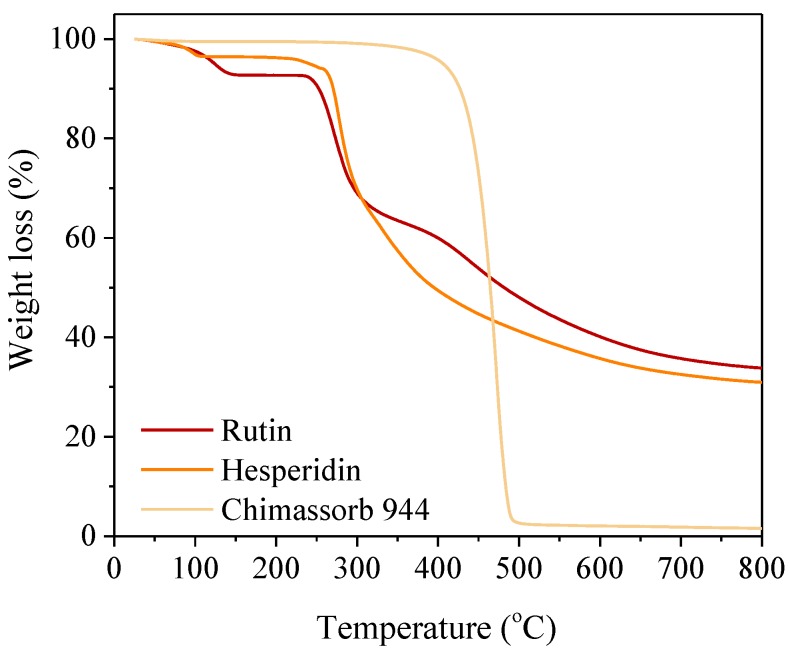
TG curves of investigated stabilizers (rutin, hesperidin, and Chimassorb 944) obtained during the temperature increase of 5 °C per minute in static air.

First, we performed a thermogravimetric analysis TG of samples of the flavonoids themselves and of the effects of flavonoids on the tested polyesters. The stability of the flavonoids themselves was extremely important from the perspective of processing polymers; it is important for the additives to be stable at processing temperatures. The results indicated that the most thermostable compound was the Chimassorb 944 industrial stabilizer; its temperature at 20% mass loss was as high as 442.50 °C. Hesperidine and rutin were characterized by a T_20_ temperature of 279–281 °C (Figure 3).

**Figure 4:**
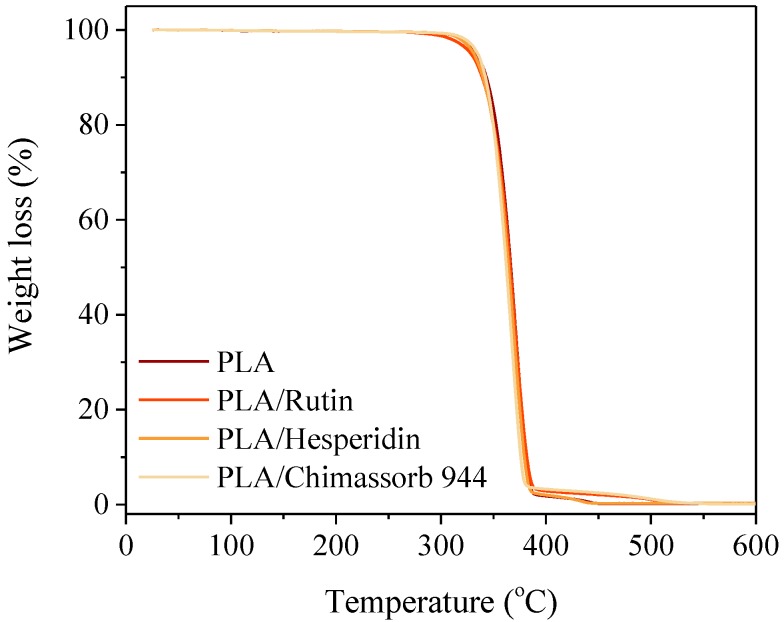
TG curves of investigated PLA stabilized with 1 phr of rutin, hesperidin, and Chimassorb 944 obtained during the temperature increase of 5 °C per minute in static air.

**Figure 5:**
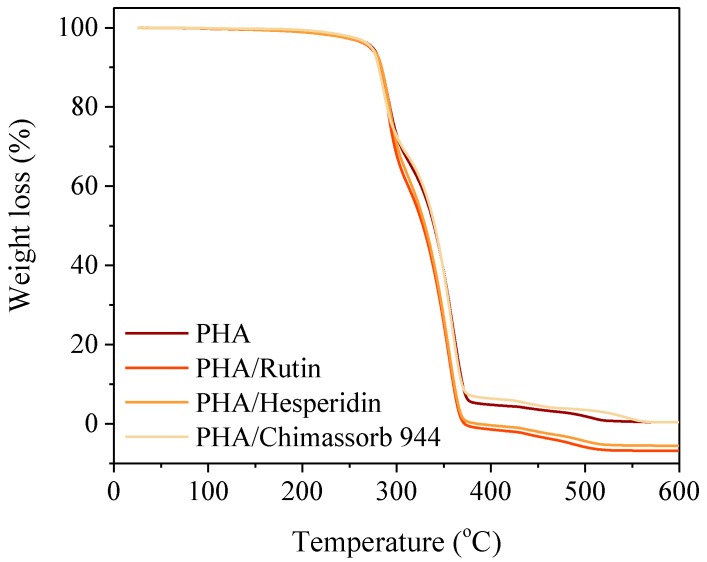
TG curves of investigated PHA stabilized with 1 phr of rutin, hesperidin, and Chimassorb 944 obtained during the temperature increase of 5 °C per minute in static air.

PLA itself was more stable with a temperature at 20% mass loss of 392 °C. Flavonoid addition did not have a marked effect on the stability of the polyesters. Rutin and hesperidine were not affected at the T_20_ and T_50_ PLA and PHA temperatures. The impact of the commercial UV absorber was identical to that of the flavonoids (Figure 4 and Figure 5).

Next, we performed DSC. The results showed that the addition of both natural and synthetic stabilizers did not affect the PLA glass transition temperature. However, it was noticeable that the oxidation temperature increased with the addition of the antioxidants. For example, hesperidine increased the oxidation temperature *T*_0_ to 27 °C. Chimassorb also raised the oxidation temperature by approximately 16–17 °C. Surprisingly, a significant reduction in PLA oxidation energy was observed (Δ*H*_0_ = 21.69 J/g) by adding rutin (Δ*H*_0_ = 11.85 J/g) and hesperidin (Δ*H*_0_ = 8.38 J/g). A reduction in oxidation energy was associated with improved stability by reducing the oxidation effect. The melting enthalpy values were lowest for the PLA/Chimassorb sample (Δ*H*_0_ = 6.07 J/g) and highest for the PLA/hesperidin sample (Δ*H*_0_ = 19.40 J/g). However, it was apparent that after antioxidant ageing, all the tested antioxidants acted in a stabilizing manner as opposed to solar ageing. After solar ageing, the PLA/Chimassorb 944 sample had the best high-stability properties (Table 1).

**Figure 6:**
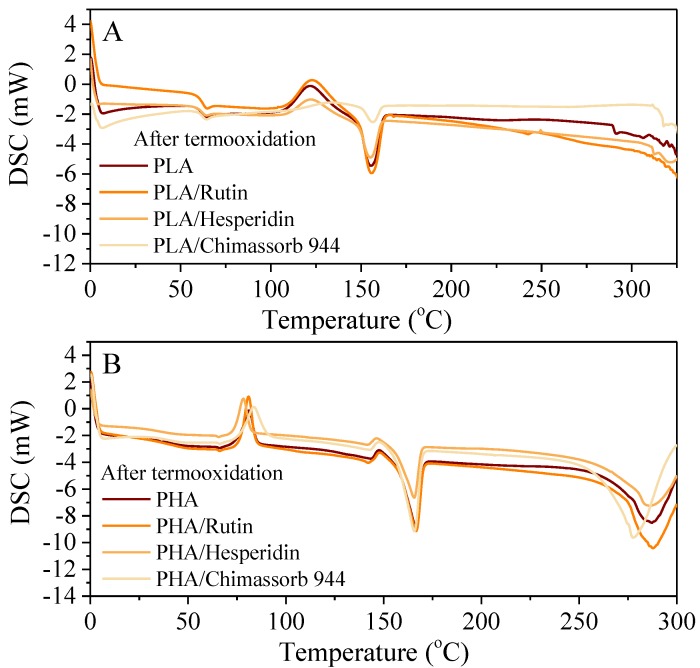
DSC curves of (**A**) PLA and (**B**) PHA stabilized with 1 phr of rutin, hesperidin, and Chimassorb 944 obtained by heating from room temperature to 300 °C.

Before ageing, the PHA reference samples and PHA with stabilizers had similar thermal characteristics. The low-molecular-mass addition was similar to PLA in terms of increasing the oxidation temperature to 44 °C with hesperidin and 30 °C with the commercial stabilizer. In contrast to PLA samples, PHA samples were more resistant to solar ageing than to long-term thermo-oxidative effects. Such results might be closely related to the various dispersions and impeded lability of stabilizers in the polyester matrixes PHA and PLA (Table 2, Figure 6).

### 3.2. Physicochemical Properties of PLA and PHA Composites with Antioxidants

Many food products are packed in polymeric materials. However, it should be noted that such polymers contain low-molecular-mass additives, which tend to migrate from the food packaging. Modern packaging should comply with the European Commission Regulation 2011 on plastic materials and articles intended to come into contact with food.

**Figure 7:**
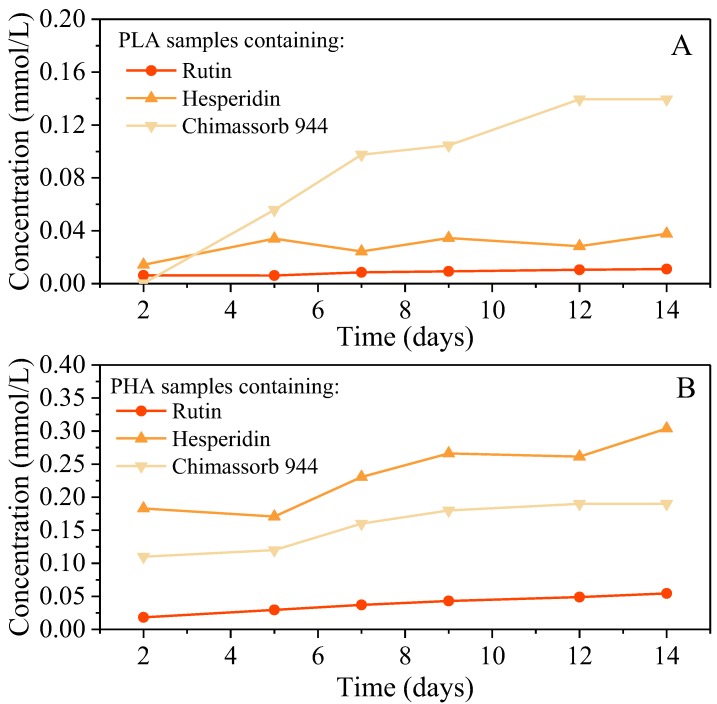
Global migration results in the simulant (ethanol) from extruded laboratory samples (**A**) PLA and (**B**) PHA.

The level of migration of low-molecular-mass antioxidant compounds into the model solution was determined using an analytical assay. In this study, ethanol as a fatty food simulant was analyzed at 24 °C. The migration process of the antioxidants was studied by UV-Vis spectroscopy because many additives absorb light in the UV region. The use of natural substances of plant origin in packaging materials is beneficial to both future consumers and manufacturers of packaging with environmentally friendly intentions. Migration after 14 days with a value greater than or equal to 0.011 mmol/L in the polylactide sample was most likely due to migration kinetics. The migration after 14 days was found to be 0.140 mmol/L. The prevalence of migration was noticeable in the PHA sample (migration after 14 days = 0.304 mmol/L), while the lowest level of migration seen for routine use was found to be 0.054 mmol/L after 14 days. The stability of all antioxidants used in PHA was much higher than when used in PLA. Taking into account the chemical composition of stabilizers, the most difficult and slowest should be HAS migration, while rutin and hesperidin have the same molecular size. This theory has not yet been confirmed in practical experiments. Thus, it can be clearly stated that innovative antioxidants have a level of migration to the environment that is similar to commercial-scale stabilizers, and they are therefore acceptable (Figure 7).

**Figure 8:**
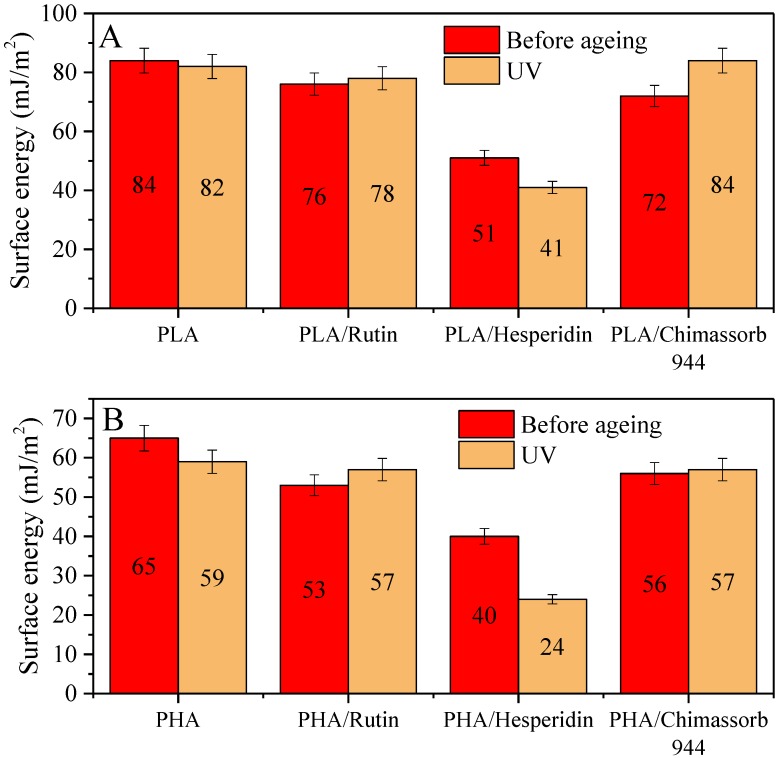
Surface free energy (mJ/m^2^) of (**A**) PLA and (**B**) PHA stabilized with rutin, hesperidin, and Chimassorb 944.

The surface properties of polymeric samples sometimes determines their susceptibility to ageing. The hydrophilicity and surface hydrophobicity were very important parameters for evaluating the oxidation processes of the polymer. Contact angle measurements of PLA were calculated based on the method proposed by Owens et al., as seen in Figure 8. Surface properties of the PLA film were evaluated by contact angle measurements using water and diiodomethane as test liquids.

The surface energy of polyesters after the addition of stabilizers was generally reduced in both PLA and PHA. The antioxidant supplement can increase the number of polar groups. PLA alone had an energy of 84 mJ/m^2^, which was reduced by 33 mJ/m^2^ upon the addition of hesperidin. Similar phenomena were observed for PHA samples with an energy of 65 mJ/m^2^, but after hesperidine addition, the value was decreased by 25 mJ/m^2^. It is likely that hesperidine is labile in polymers and migrates to the surface where it alters the surface properties through the presence of polar groups. The other applied stabilizers did not greatly change the surface properties of the tested polyesters.

**Figure 9:**
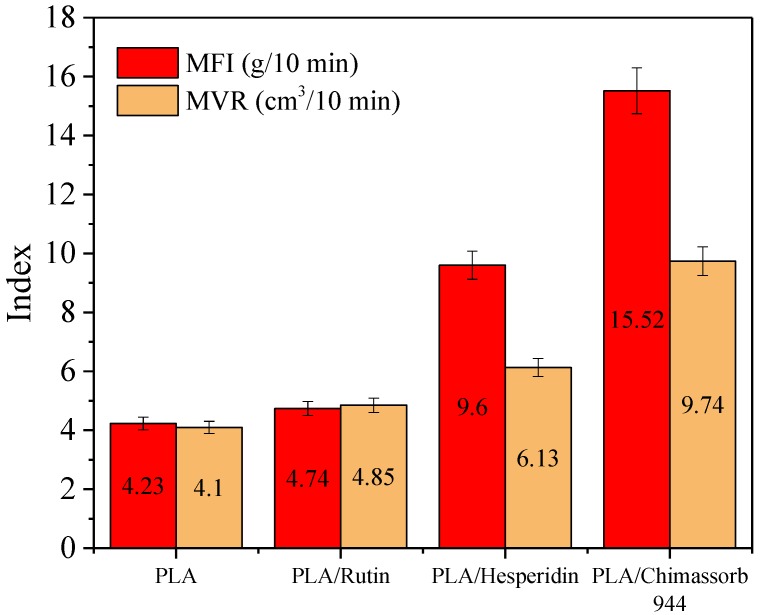
Melt flow index (MFI) and melt volume rate (MVR) of PLA stabilized with rutin, hesperidin, and Chimassorb 944.

The impact of stabilizers on the flow index of the PLA polyester was also investigated. PLA itself had an MFI of 4.23 g/10 min and an MVR of 4.23 cm^3^/10 min. The use of additives in PLA resulted in almost a doubling of both the MFI and MVR ratios. Rutin did not affect the change in flow rate indexes of polylactide, while hesperidine worked more plastically than the commercial stabilizer (Figure 9).

**Figure 10:**
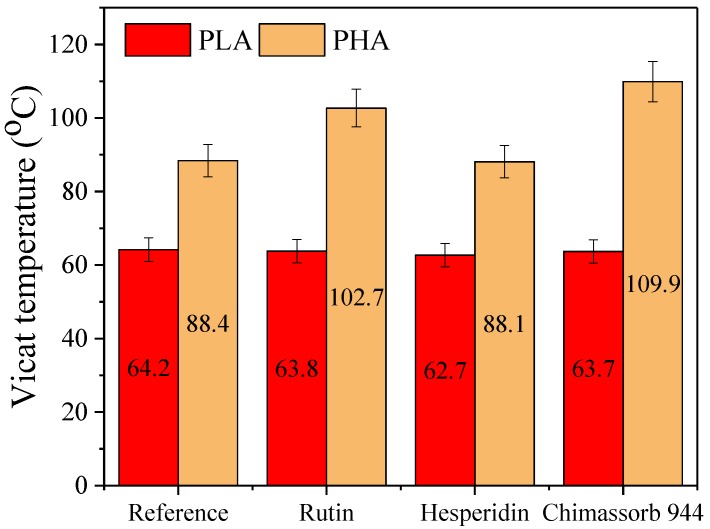
Vicat softening temperature of PLA and PHA stabilized with rutin, hesperidin, and Chimassorb 944.

The results of the softening temperature experiment, determined according to the Vicat method, indicated that the addition of both flavonoid and amine antioxidants did not significantly affect the plastic softening temperature. It can be argued that PHA is much harder and has a higher softening temperature compared to PLA. Temperatures of the polyhydroxyalkanoate PHB reference and PHB loaded with hesperidin were similar, but a higher change was observed for Chimassorb (Figure 10).

**Figure 11:**
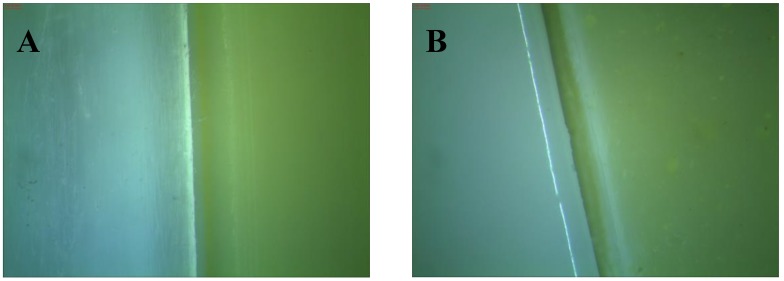
Photographs of samples with flavonoids: (**A**) PLA and PLA/rutin and (**B**) PHA and PHA/rutin.

**Figure 12:**
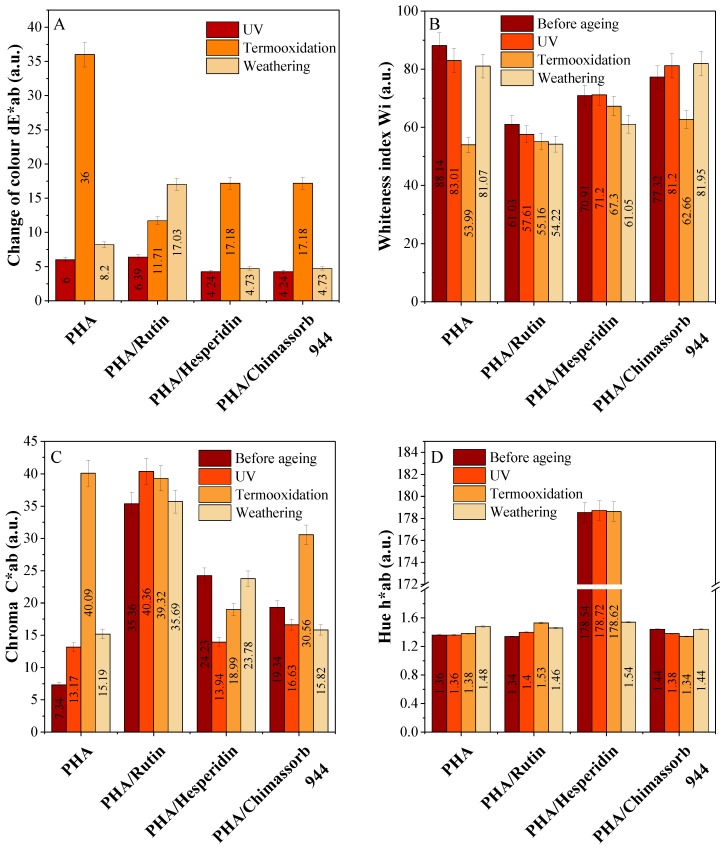
Color difference (dE*ab) (**A**), change of whiteness index (**B**), chroma (**C**) and hue (**D**) of PHA composites stabilized with 1 phr of rutin, hesperidin, and Chimassorb 944 after UV ageing and weathering was measured according to the Cie-Lab color scale. Coordinate a* green/red color component (a* > 0 red, a* < 0 green) and b* blue/yellow color component (b* > 0 yellow, b* < 0 blue).

**Figure 13:**
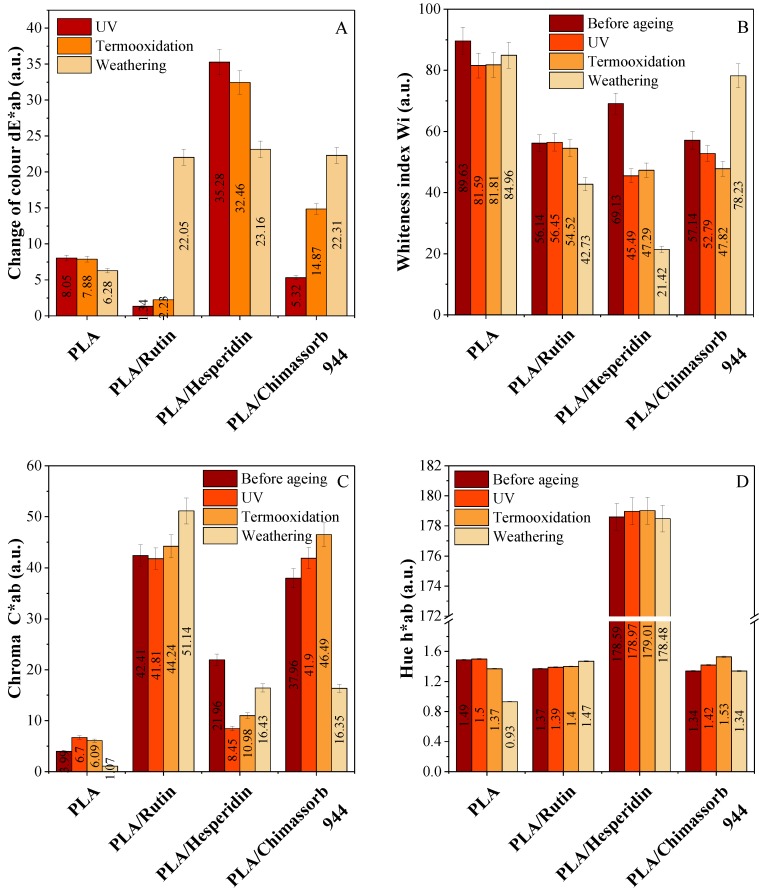
Color difference (dE*ab) (**A**), change of whiteness index (**B**), chroma (**C**) and hue (**D**) of PLA composites stabilized with 1 phr of rutin, hesperidin and Chimassorb 944 after UV ageing and weathering was measured according to the Cie-Lab color scale. Coordinate a* green/red color component (a* > 0 red, a* < 0 green) and b* blue/yellow color component (b* > 0 yellow, b* < 0 blue).

Color is one of the most important parameters that can significantly change the visual properties of polyester samples. The addition of natural flavonoid antioxidants gave a beautiful and distinctive color to the polyester samples. Figure 11 shows an exemplary photograph of samples with flavonoids. The photos were taken with the Leica stereoscopic microscope. The software Optaview was used to analyze and process photos of selected samples. The pictures show samples at 43× magnification. These flavonoids act as natural pigments and dyes in plants. Using colorimetric analysis, the UV-Vis spectrophotometer could detect color changes that occurred not only under the influence of natural antioxidants, but also to a great extent under the influence of climatic factors. The measurements by the Cie-Lab used the coordinates in a uniform color space consisting of L* and chromaticity indices a* and b*. In the case of PHA, the chroma parameter and brightness were the most affected by ageing. Significant color changes (dE*ab) were observed after thermal ageing combined with oxidation. Surprisingly, when exposed to solar radiation, the sample exhibited limited color change as detected by this UV instrument (chroma, hue, and whiteness index). The reason for this might have been too small a dose of UV ageing and weathering. In contrast, the polylactide samples significantly changed color in comparison to the PHA samples (Figure 12 and Figure 13).

The degradable PLA and PHA polymers used, although different, have been tested for the effect of plant-derived additives on their stability. Interesting research results have been obtained, which indicates the improvement of polyester resistances to the oxidation, and what is associated with it may suggest that they will have a longer “life time” span without losing their degradability after the end of their service life. The addition of natural antioxidants means that the produced materials can be considered as environmentally friendly. Interesting packaging materials have been obtained that are characterized by a specific lifetime and are also colored thanks to the addition of flavonoids. The color of such materials can be used in many applications, such as in the labeling of packaged food.

## 4. Conclusions

This study revealed that the stabilization of polyesters by the addition of plant-derived substances was a successful and effective process. The addition of rutin and hesperidin significantly increased the resistance to oxidation of both PLA and PHA. The presence of hydroxyl groups in the flavonoids’ structure gave them excellent antioxidant properties.

The reaction of the OH groups during the oxidation reaction was indicated by a color change, and therefore the polymer ageing processes could be monitored in a controlled way. In addition, flavonoids are natural, commonly occurring substances that are widespread in the plant world. With the growth of plastics consumption, it is important to seek out and study new environmentally friendly stabilizers. The combination of polymers obtained from renewable raw materials with the addition of plant substances is an interesting alternative to existing materials.

## Figures and Tables

**Table 1:** The Differential Scanning Calorimetry DSC analysis of PLA samples.

Sample	*T*_g_ (°C)	Δ*H*_cc_ (J/g)	*T*_cc_ (°C)	Δ*H*_m_ (J/g)	*T*_m_ (°C)	Δ*H*_0_ (J/g)	*T*_0_ (°C)
**Before Ageing**							
PLA	59.66	18.94	108.10	16.82	148.68	21.69	229.41 298.22
PLA/Rutin	59.15	12.20	111.33	12.54	148.97	11.85	245.79 305.38
PLA/Hesperidin	59.40	19.45	107.25	19.40	148.35	8.38	256.29 308.72
PLA/Chimassorb 944	59.81	8.13	114.90	6.07	151.04	26.52	246.22 19.73
**After Thermo-Oxidation Ageing**							
PLA	58.37	27.59	107.76	23.67	148.43	19.08	223.76 290.52
PLA/Rutin	59.58	26.83	108.43	21.42	148.94	2.36	242.58 271.04
PLA/Hesperidin	58.51	24.34	108.27	21.44	148.32	0.94	248.57 250.67
PLA/Chimassorb 944	58.67	7.57	111.92	5.61	150.63	7.03	306.02 313.25
**After Weathering Ageing**							
PLA	58.50	27.92	107.92	23.03	149.04	26.45	259.74 293.48
PLA/Rutin	58.43	30.56	105.52	25.06	147.79	10.84	237.57 300.43
PLA/Hesperidin	42.02	34.63	92.66	37.73	139.99	17.14	230.06 270.01
PLA/Chimassorb 944	58.44	11.04	111.85	8.79	150.46	15.72	271.96 316.19

*T*_g_ = glass transition temperature, Δ*H*_cc_ = enthalpy of crystallization, *T*_cc_ = crystallization temperature, Δ*H*_m_ = enthalpy of melting, *T*_m_ = melting temperature, Δ*H*_0_ = enthalpy of oxidation, *T*_0_ = initial and final oxidation temperature.

**Table 2:** The Differential Scanning Calorimetry DSC analysis of PHA samples.

Sample	*T*_g_ (°C)	Δ*H*_cc_ (J/g)	*T*_cc_ (°C)	Δ*H*_m_ (J/g)	*T*_m_ (°C)	Δ*H*_0_ (J/g)	*T*_0_ (°C)
**Before Ageing**							
PHA	36.79	15.36	76.75	(1) 6.53 (2) 33.93	(1) 127.85 (2) 156. 67	9.66	199.27 260.33
PHA/Rutin	39.67	13.37	81.51	(1) 5.76 (2) 28.93	(1) 128.52 (2) 156.59	1.31	235.12 255.89
PHA/Hesperidin	36.57	13.87	77.35	(1) 6.15 (2) 32.57	(1) 128.41 (2) 157. 36	20.63	243.57 275.98
PHA/Chimassorb 944	37.37	16.66	78.47	(1) 3.86 (2) 31.50	(1) 126.52 (2) 156. 49	1.71	229.18 251.18
**After Thermo-Oxidation Ageing**							
PHA	35.14	14.73	75.02	(1) 6.09 (2) 35.57	(1) 123.17 (2) 156.37	2.12	209.36 251.44
PHA/Rutin	36.93	17.50	76.49	(1) 9.48 (2) 36.05	(1) 132.57 (2) 156. 43	3.68	195.74 255.11
PHA/Hesperidin	35.30	16.50	73.33	(1) 4.95 (2) 36.27	(1) 128.93 (2) 155.68	19.00	201.67 264.40
PHA/Chimassorb 944	37.12	15.85	76.17	(1) 4.44 (2) 34.74	(1) 125.25 (2) 155.88	1.61	207.06 243.30
**After Weathering Ageing**							
PHA	27.85	13.54	71.15	(1) 4.56 (2) 34.16	(1) 129.94 (2) 153.67	4.23	185.68 243.37
PHA/Rutin	26.24	14.41	71.94	(1) 4.44 (2) 35.67	(1) 126.63 (2)154.26	9.52	204.71 262.03
PHA/Hesperidin	35.38	11.44	63.90	(1) 0.12 (2) 30.33	(1) 100.29 (2) 130.63	7.36	200.69 249.40
PHA/Chimassorb 944	30.35	8.29	67.09	(1) 3.08 (2) 28.94	(1) 123.08 (2) 153.01	8.13	204.04 251.20

*T*_g_ = glass transition temperature, Δ*H*_cc_ = enthalpy of crystallization, *T*_cc_ = crystallization temperature, Δ*H*_m_ = enthalpy of melting, *T*_m_ = melting temperature (first melting endotherm (1) and second melting endotherm (2)), Δ*H*_0_ = enthalpy of oxidation, *T*_0_ = initial and final oxidation temperature.
